# Deep Hierarchical Ensemble Model for Suicide Detection on Imbalanced Social Media Data

**DOI:** 10.3390/e24040442

**Published:** 2022-03-23

**Authors:** Zepeng Li, Jiawei Zhou, Zhengyi An, Wenchuan Cheng, Bin Hu

**Affiliations:** 1 School of Information Science and Engineering, Lanzhou University, Lanzhou 730000, China; lizp@lzu.edu.cn (Z.L.); zhoujw20@lzu.edu.cn (J.Z.); anzhy20@lzu.edu.cn (Z.A.); chengwch20@lzu.edu.cn (W.C.); 2 Institute of Engineering Medicine, Beijing Institute of Technology, Beijing 100081, China; 3 CAS Center for Excellence in Brain Science and Institutes for Biological Sciences, Shanghai Institutes for Biological Sciences, Chinese Academy of Sciences, Shanghai 200000, China

**Keywords:** social media, suicide ideation detection, deep neural network, imbalanced data, Sina Weibo, China

## Abstract

As a serious worldwide problem, suicide often causes huge and irreversible losses to families and society. Therefore, it is necessary to detect and help individuals with suicidal ideation in time. In recent years, the prosperous development of social media has provided new perspectives on suicide detection, but related research still faces some difficulties, such as data imbalance and expression implicitness. In this paper, we propose a Deep Hierarchical Ensemble model for Suicide Detection (DHE-SD) based on a hierarchical ensemble strategy, and construct a dataset based on Sina Weibo, which contains more than 550 thousand posts from 4521 users. To verify the effectiveness of the model, we also conduct experiments on a public Weibo dataset containing 7329 users’ posts. The proposed model achieves the best performance on both the constructed dataset and the public dataset. In addition, in order to make the model applicable to a wider population, we use the proposed sentence-level mask mechanism to delete user posts with strong suicidal ideation. Experiments show that the proposed model can still effectively identify social media users with suicidal ideation even when the performance of the baseline models decrease significantly.

## 1. Introduction

According to statistics from the World Health Organization (WHO) [1], 703,000 people committed suicide worldwide in 2019, and suicide has become a serious worldwide problem. Through early identification of individuals with suicidal ideation, suicide can be effectively prevented. China is the most populous country in the world. The huge population base determines that the Chinese potential suicide population is relatively large. Therefore, it is necessary to conduct suicide detection for Chinese. Commonly used suicide detection methods are mainly clinical diagnosis by doctors or the use of various scales, such as Suicide Probability Scale [2], the Suicidal Ideation Questionnaire [3], and the Suicidal Affect–Behavior–Cognition Scale (SABCS) [4]. Clinical diagnosis can effectively identify the patient’s suicidal ideation, but the experienced clinician is very rare and only a small number of patients will actively seek help from a doctor. For fear of discrimination or disclosure of privacy, suicidal individuals may conceal their true situation when they realize that they are under supervision. Therefore, methods such as questionnaires and clinical diagnosis are not suitable for screening a large number of potential suicide-prone individuals.

In recent years, with the continuous expansion of the Internet, social media has also developed rapidly. Billions of people from all over the world express their opinions on social media every day. Compared with the real world, the Internet has inherent concealment, so people tend to express their true feelings on social media. At the same time, most of the data on social media is public, which makes it possible to use the data for suicide detection. Some works used data on social media such as Twitter, Reddit, Sina Weibo, etc., for suicide detection [5,6,7]. These undisturbed suicide detection methods would not cause individual resistance, and the collected data can better reflect their true emotions.

However, there are some problems in using data on social media for suicide detection. One of the important problems is data imbalance. According to data released by WHO [1], in 2019, the age-standardized suicide rate in China was 6.7 per 100,000 people. We cannot know the specific number of people with suicidal ideation, but it can be inferred that the proportion of individuals with suicidal ideation is extremely low, so the data obtained from social media is often imbalanced, and only a small number of users actually have suicidal ideation. At present, the research on suicide detection using social media data mainly depends on machine learning or deep learning methods. Training the classification model directly with imbalanced data will seriously affect the performance of the model, so it is essential to provide measures to deal with this problem. There are some works dedicated to solving the imbalance problem in text classification, such as improving the traditional oversampling [8] and undersampling [9] methods, or modifying the loss function of the model [10], etc. However, there are few works focusing on the data imbalance problem on Chinese social media suicide detection.

In most suicide detection studies, the data of users with suicidal ideation usually comes from relatively private sub-forums or ‘tree holes’ on social media. The so-called ‘tree hole’ on Weibo is a place where people anonymously share their secrets with others [11]. These users would post posts expressing strong pessimism or negative emotions in tree holes influenced by others. Many users with suicidal ideation may not express suicidal thoughts in tree holes. Suicide detection using data containing tree holes posts will omit these users. Therefore, the suicide detection model trained by using data in tree holes is difficult to be used widely. Some studies used word-level mask mechanism to mask some keywords [12], and still achieved better classification results. Similarly, we hope that even if the posts with obvious suicidal ideation in the tree holes is removed, the model can still effectively detect users’ suicidal ideation from their remaining posts, thereby making the suicide detection task more universal.

Another common problem in the field of Chinese suicide detection is the lack of public datasets with high credibility. The existing suicide detection datasets based on social media are mainly in English. Reddit, an English social platform, has a sub-forum called ‘Suicide Watch’, in which many Reddit users would post posts with suicidal thoughts. Therefore, most data for suicide detection studies in English text comes from this forum [13,14,15]. There are also some studies obtained data from Chinese social media, such as Weibo, for suicide detection. However, due to the privacy policies of social media platforms and the ethical issues that may arise from suicide detection research, researchers must be very careful to disclose the data. The lack of public datasets greatly limits the development of suicide detection study.

To cope with the above problems, we propose a **D**eep **H**ierarchical **E**nsemble model for **S**uicide **D**etection (**DHE-SD**) based on a hierarchical ensemble strategy, which can effectively detect a low proportion of suicide-prone users on social media. Classical ensemble methods need more than half of the base classifiers to get the final correct results, while the hierarchical ensemble strategy may still give the correct results when less than half of the base classifiers are correct. On the other hand, we construct a Chinese social media suicide detection dataset based on Weibo to alleviate the lack of relevant datasets. As an imbalanced dataset, it partially reveals a real world phenomenon that the number of suicide-prone individuals is less than normal individuals. Experiments show that the proposed model achieves considerable results on both our dataset and a public Sina Weibo dataset (https://github.com/bryant03/Sina-Weibo-Dataset (accessed on 20 March 2022)). In addition, we use the proposed sentence-level mask mechanism to delete user posts in the ‘tree hole’. The results indicate that the proposed model still has fine performance. In this case, the trained model can be more easily extended to more general suicide detection tasks.

In general, our work has the following contributions:

- To address the problem of data imbalance in the suicide detection field, we propose a deep learning model based on a hierarchical ensemble strategy, which can effectively identify suicide-prone users on social media.
- Due to the lack of a Chinese social media suicide detection dataset, we provide a dataset based on the Weibo platform. This dataset can promote further research related to suicide in the fields of computer science and psychology.
- In order to determine the effectiveness of the DHE-SD model after deleting tree hole posts with strong suicidal ideation, we use the proposed sentence-level mask mechanism to delete these posts. The results show that, in this case, compared with the baseline, the proposed model can also effectively detect suicide-prone individuals.

The rest of this paper is organized as follows. In Section 2, we review the related work in the field of suicide detection. Section 3 details the proposed method and model. In Section 4, we describe the suicide detection dataset based on Weibo. We introduce the experimental setup and process in Section 5. In Section 6, we discuss and analyze the experimental results. Section 7 concludes the work with future research directions.

## 2. Related Work

In this section, we briefly introduce the research progress in the field of suicide detection. We introduce the research methods of suicide detection using classical machine learning and deep learning. Then, in view of the data imbalance problem in the field of text classification and suicide detection, we introduce the development status of related research. Finally, we elaborate on the research of the mask problem.

The rapid development of machine learning and deep learning makes it possible to detect suicidal ideation on social media more efficiently. Ji et al. [16] classified and introduced the machine learning and deep learning methods of suicide ideation detection. Masuda et al. [17] applied logistic regression to classify the suicide group and the control group according to the social behavior characteristics of users. Desmet et al. [18] constructed a suicide note analysis method using support vector machines, which can be used for fine-grained emotion classification. Huang et al. [19] used a rule-based method to develop a real-time suicide ideation detection system by combining machine learning and psychological knowledge, then deployed it on the Weibo platform. Kim [20] used a convolutional neural network to classify text for the first time. Joulin et al. [21] proposed the FastText model, which can speed up model training under the premise of high-precision classification. Johnson et al. [22] proposed a low-complexity deep convolutional neural network for text classification. This model can effectively obtain a representation of text long-distance connections. In order to detect suicidal ideation in private chat rooms, Ji et al. [23] proposed a model aggregation method for updating neural networks. Benton et al. [24] used a multi-task learning framework to predict suicidal ideation by predicting the gender of users as an auxiliary task. Cao et al. [7] improved the detection performance by constructing the social knowledge graph of users on social media. Sinha et al. [25] used a stack ensemble method for suicide detection. In addition to text information, this work also used time context and social network information of users. Ren et al. [26] proposed a cumulative emotion model that can classify suicide blog streams. Zhao et al. [27] used the D-CNN model to detect users with depression in Weibo’s tree holes. Matero et al. [28] proposed a dual-context model using hierarchical attention RNN and BERT to identify suicidal ideation on social media. Sawhney et al. [29] proposed a hierarchical attention model called SISMO, which improved the level of fine-grained suicide detection through soft probability distribution.

Data imbalance is a very important problem in the field of text classification, which greatly limits the performance of the widely used text classification methods based on machine learning and deep learning. One strategy to deal with this problem is to sample the data so that the number of samples in each category is similar. Tang et al. [8] performed multiple undersampling on the data and trained the Bert classifier using the obtained dataset. SMOTE [30] is a classic oversampling method in the field of machine learning, but applying this method directly to text may lose the semantic information. Li et al. [9] proposed a new oversampling method that directly used the original data to generate new synthetic text. However, these two kinds of methods have certain shortcomings. Oversampling may easily lead to overfitting of the model, while undersampling may lose important information of the sample. Another strategy focuses on modifying the algorithm, where cost-sensitive learning is one of the representative methods. Wang et al. [10] obtained the cost matrix of the sample according to the Bayesian decision theory, combined it and the cross-entropy loss, and proposed a new loss function. There are also some works that used genetic algorithms and differential evolution algorithms to get the cost matrix [31,32]. Lodkaew et al. [33] combined two loss functions with better performance to improve the accuracy of text classification using deep neural networks. In addition, some studies applied feature selection to solve the problem of data imbalance in text [34,35,36]. Most of these works use feature engineering to select features that can better distinguish minority samples. However, there are few studies that specifically address the problem of data imbalance in the field of suicide detection, and most of the existing works only use simple oversampling or undersampling methods [37,38].

Aiming at the mask problem, Chiong [12] used machine learning methods to classify depression based on social media posts. Experimental results showed that these methods could effectively detect depression when keywords such as ‘depression’ and ‘diagnosis’ are masked in datasets. Preoţiuc et al. [39] constructed a complaints dataset obtained from Twitter, in which the positive samples came from tree holes, and performed sentence-level classification by combining language features and domain adaptive methods. Cao et al. proposed a suicide dataset based on Weibo in [7], including 3652 suicide-prone users and 3677 users without suicidal ideation. They constructed a knowledge graph based on the user’s attributes and post content, and used GNN with attention mechanism to detect individuals with suicidal ideation on social media. Cao [40] studied implicit and anti-real suicidal expressions in Weibo. They combined general domain information with specific information in the suicide domain, and proposed a two-layer attention mechanism model. The datasets proposed by Cao et al. contained data of tree holes.

## 3. Methodology

In recent years, methods based on deep neural networks have made great progress in sentiment analysis. In this section, a deep learning method based on hierarchical ensemble is proposed to detect the suicidal ideation of Weibo users. Among a large number of social media users, only a small number of users have suicidal ideation. A small number of users with suicidal ideation would express their suicidal ideation in the tree hole, so if only the data of tree hole is analyzed, it is impossible to find other suicide-prone users who do not post in the tree hole. The goal of this paper is to identify users with suicidal ideation who account for a small number of social media users based on their open posts, and also try to identify these users without using tree hole data.

### 3.1. Problem Definition

For user $u_{i}\in U,i=1,2,\ldots ,x$, where *x* represents the total number of users. The complete posting sequence of each user is $T_{i}=\left\{t_{1},t_{2},\ldots ,t_{y_{i}}\right\}$, where $y_{i}$ represents the total number of posts posted by user *i*. The post sequence after using the sentence-level mask mechanism to delete the post in the tree hole is $T_{i}^{\prime }=\left\{t_{1^{\prime }},t_{2^{\prime }},\ldots ,t_{y_{i}^{\prime }}\right\}$, where $y_{i}^{\prime }$ represents the number of posts after deleting user *i*’s tree hole content, and $y_{i}^{\prime }\leq y_{i}$. Each user is given a label ${\bar{y}}_{l}\in \left\{\mathrm{suicidal},\mathrm{non}-\mathrm{suicidal}\right\},i=1,2,\ldots ,x$.

### 3.2. Deep Hierarchical Ensemble Model for Suicide Detection

**Figure 1:**
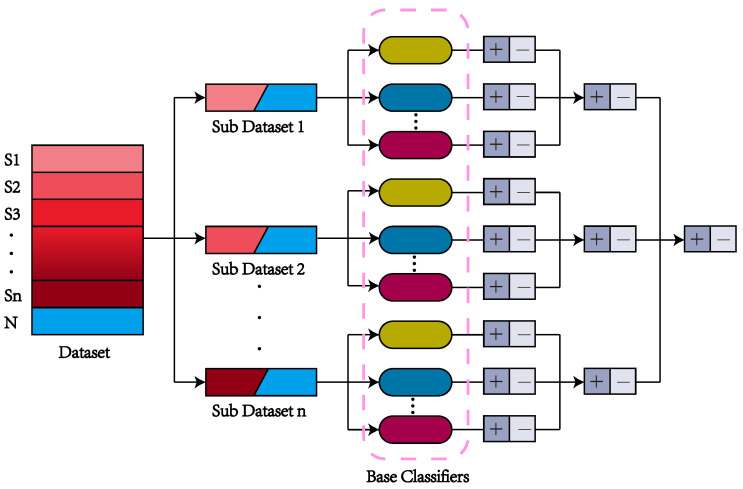
The architecture of DHE-SD model.

On social media, only a small number of users have suicidal ideation, and their posts account for a small proportion of the total posts on social media. The data for suicide detection on social media is extremely imbalanced. Aiming at this problem, the paper proposes a hierarchical ensemble strategy. Based on this strategy, we propose a DHE-SD model; the model structure is shown in Figure 1.

Specifically, the method divides the imbalanced dataset *D* into n balanced sub-datasets $D_{1},D_{2},\ldots ,D_{n}$ firstly, so that $D=D_{1}\cup D_{2}\cup \ldots \cup D_{n}$, and then uses the sub-datasets to train base classifiers. We train a series of base classifiers $C_{p}=\left\{c_{p}^{1},c_{p}^{2},\ldots ,c_{p}^{q},\ldots ,c_{p}^{m}\right\}$ for each sub-dataset $D_{p}\subseteq D$, where *m* represents the number of base classifiers.

For user $u_{i}$ in the test set, concatenating his/her posting sequence $T_{i}$ as input of the classifiers:

(1) $$d_{i}=\left[t_{1}∥t_{2}∥\ldots ∥t_{y_{i}}\right]$$

Using the trained multiple base classifiers to classify user $u_{i}$, respectively, and obtaining the prediction results of each base classifier:

(2) $$r_{p}^{q}\left(u_{i}\right)=c_{p}^{q}\left(d_{i}\right)$$

where $\left\{r_{p}^{1},r_{p}^{2},\ldots ,r_{p}^{m}\right\}$. Then get the ensemble result of the base classifier trained by the sub-dataset $D_{p}$:

(3) $$r_{p}\left(u_{i}\right)=E\left(r_{p}^{1}\left(u_{i}\right),r_{p}^{2}\left(u_{i}\right),\ldots ,r_{p}^{m}\left(u_{i}\right)\right)$$

where *E* represents ensemble operation. We integrate the ensemble results $r_{p}\left(u_{i}\right)$ of the base classifier $C_{p}$ trained with each sub-dataset $D_{p}$ on the test set again to obtain the final results:

(4) $$r\left(u_{i}\right)=E\left(r_{1}\left(u_{i}\right),r_{2}\left(u_{i}\right),\ldots r_{n}\left(u_{i}\right)\right)$$

**Figure 2:**
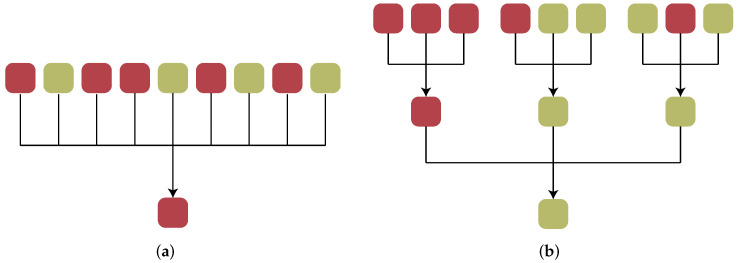
Hierarchical Ensemble. (**a**) An example of a classical ensemble method, and (**b**) an example of a hierarchical ensemble method, where red rectangle represents the base classifier with wrong classification result, and green rectangle represents the base classifier with correct classification result. Taking nine base classifiers as an example, the classical ensemble method cannot give correct prediction results when the number of correct base classifiers is less than half of the total number of classifiers. Through the continuous combination of base classifiers, the hierarchical ensemble method can still give correct prediction results even if the number of correct classifiers is less than half.

Figure 2 intuitively shows the superiority of our proposed hierarchical ensemble strategy compared to classical ensemble methods.

### 3.3. Sentence-Level Mask

**Figure 3:**
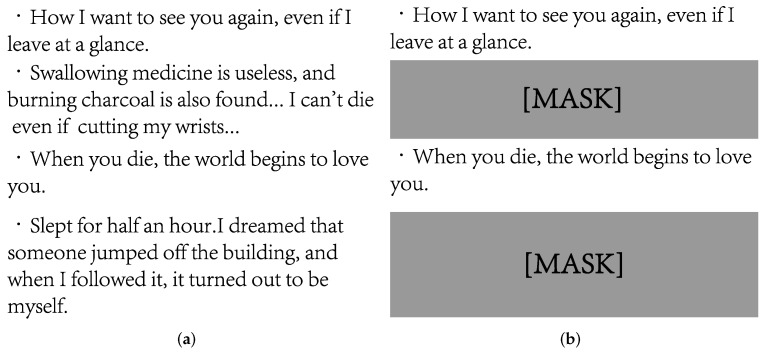
An example of sentence-level mask mechanisim. (**a**) User posts before mask, and (**b**) User posts after mask.

Existing works usually use tree hole data that contains strong suicidal ideation. However, this may simplify suicide detection to a sentiment classification task, and it is hard to distinguish whether an individual actually has suicidal ideation. In addition, many potential suicidal individuals never post in these tree holes, and it is difficult to discriminate between them and previous methods. In order to alleviate the above problems, we propose a novel sentence-level mask mechanism. Specifically, this mechanism can delete posts with obvious suicidal ideation in the tree hole, so that the trained model can be more widely used in suicide detection tasks. An example of the sentence-level mask mechanism is shown in Figure 3.

### 3.4. Deep Learning Model

Classical machine learning models, such as SVM, classify data based on sample features, or use methods such as clustering to find users with suicidal ideation. These methods need to extract features manually, and the recognition accuracy is relatively low. Deep neural network methods can automatically learn feature representations and have better classification performance.

- TextCNN [20] is a shallow model. First, multiple convolution kernels are used to convolute words to obtain feature vectors. Then, each feature vector is mapped to a number through the max-pooling layer, and the pooling result is concatenated into a vector, and finally the classification result is obtained through a fully connected layer.
- FastText [21] is a simple and effective text classification model. Different from classical text classification methods, FastText superimposes word embeddings to obtain document vectors, and word embedding itself can measure the semantic similarity of words.
- DPCNN [22] utilizes multiple convolution and pooling modules to form a pyramidal convolutional neural network, which uses the max-pooling layer to compress feature length after convolution. Because the DPCNN network is deep, residual connections are added to the model to alleviate the vanishing gradient and exploding gradient problem.

## 4. Datasets

The sensitivity of the suicide detection field and the privacy of the required data lead to the lack of reliable public datasets. As for suicide detection on Chinese social media, there are few relevant datasets available for research. Therefore, we construct a dataset based on Weibo that can be used for Chinese suicide detection research, and desensitized relevant private content.

### 4.1. Dataset Construction

As the largest Chinese social platform, Weibo (https://weibo.com/ (accessed on 20 March 2022)) has more than 500 million monthly active users. Similar to the sub-forum of the social platform Reddit, Weibo has the ‘SuperTopic’ module, and each user can post in it. Since depression patients usually have serious suicidal ideation, we choose to acquire suicide-prone users in ‘Depression SuperTopic’. We identify suicide-prone users and normal users through manual annotating, which is completed by three experienced psychological experts. Specifically, the screening criteria for suicide group data is that users have posted posts with suicidal ideation in ‘Depression SuperTopic’, including revealing suicidal ideation or indicating that he/she has committed suicidal behavior. In contrast, the selection criteria for the normal group data is that all posts on the user’s homepage do not express clear suicidal ideation, even if the posts contain words related to suicide. Moreover, if the user posts social news, lyrics or poems that contains suicide keywords, we also include him/her in the normal group.

After screening, we get a total of 2019 users with suicidal ideation. In order to achieve user-level classification, we obtain all Weibo posts of these users from 1 July 2020, to 30 June 2021. Finally, we get 164,854 original Weibo posts. Similarly, we randomly acquire 2997 users as the normal group, and obtain all the posts of each user in the same period. There are 489,543 unprocessed posts in the normal group.

We use the data preprocessing method proposed in Section 4.2 to preprocess the acquired suicidal group data. We construct a dataset called **S**uicidal **W**ith**O**ut **M**ask (**SWOM**) based on these data. The dataset contains 134,260 posts of 1606 suicide-prone users. We perform the same preprocessing operation on the normal group, and finally construct a dataset called Normal, which contains 429,076 posts of 2915 normal users. We divide users into training set, validation set and test set in the ratio of 8:1:1. Specifically, the training set contains posts of 1284 suicide-prone users and 2333 normal users. The validation and test sets are composed of posts of 161 suicide-prone users and 291 normal users, respectively.

We use the sentence-level mask mechanism proposed in Section 3.2 to mask the content synchronized by users from ‘Depression SuperTopic’ in the acquired suicidal group data, and preprocess all the masked data, which makes each user’s Weibo content look more normal and indistinguishable. Based on the preprocessed data, we construct a dataset called **S**uicidal **W**ith **M**ask (**SWM**) which contains 100,286 posts of 1606 suicide-prone users. When training the DHE-SD model, we also sample the normal users in the training set and the validation set, and get three subsets equal to the number of suicide-prone users. The details of the three final datasets are shown in Table 1.

Table 2 shows post examples of users in the three datasets we constructed, in which the examples of SWOM and SWM dataset are the same user.

In order to explore the differences between the three datasets, we conduct word frequency statistics on all posts of users in each dataset. Then, we select the most representative words in each dataset, which can distinguish each other significantly. The results are shown in Table 3. It can be seen from Table 3 that the SWOM dataset includes more negative emotional words, such as ‘die’ and ‘uncomfortable’, than the other two datasets. After the sentence-level mask is performed, the representative words in the SWM dataset are relatively similar to the high-frequency words of normal users, which causes the classification task to be more difficult.

### 4.2. Data Preprocessing

We preprocess user posts in the following steps: (1) Delete the posts that are automatically generated by the system. (2) Delete the URL links that appear in the posts. (3) Delete other usernames mentioned with the ‘@’ symbol. (4) Delete the emoticons that appear in posts. (5) Delete meaningless keywords similar to ‘sharing pictures’ in the body of Weibo posts. (6) Build an administrative division list to match and delete the location information that appears in posts. (7) Use regular expressions to delete all ‘SuperTopic’ titles. (8) Delete users with too few remaining posts.

### 4.3. Public Dataset

We also use the Sina Weibo dataset constructed by Cao et al. [7]. The dataset contains all the Weibo information of 3652 users with suicidal ideation and 3677 randomly selected normal users. In order to prove the effectiveness of the proposed model, we randomly delete 1826 suicide-prone users to make the dataset have a similar distribution with the dataset we proposed. In the end, the training set contains all the posts of 3072 normal users and 1554 users with suicidal ideation, the validation and test sets contain the posts of 294 normal users and 155 suicide-prone users, respectively. Similarly, we sample the normal users in the training set and the validation set, and get three subsets equal to suicide-prone users, so that they can be sent into the DHE-SD model.

## 5. Experiments

### 5.1. Experimental Setup

The dataset used in the experiment contains 1606 suicide-prone individuals and 2915 normal individuals. Because we are performing user-level suicide detection, we concatenate all the user’s posts into a long text, and then use the Chinese word segmentation tool ‘jieba’ for word segmentation (https://github.com/fxsjy/jieba (accessed on 20 March 2022)). Note that ‘jieba’ is adapted in the general field. In order to improve the accuracy of word segmentation, we extend the dictionary of ‘jieba’ by adding words related to treatment drugs and methods for mental illnesses such as depression and anxiety. We initialize the word vector randomly, and finally use the word vector corresponding to the word in the user’s post as the input of the model.

All models are implemented using the PyTorch framework, a single GPU (Nvidia Tesla V100) is used when training the model and random search is used to determine the optimal parameters of the model. For TextCNN and DPCNN, we train for 20 epochs, while with FastText for 50 epochs. At the same time, in order to prevent the model from overfitting, we use the early stopping strategy. In addition, the batch size of every model is set to 128.

### 5.2. Experimental Process

In this paper, we select three models as base classifiers. Correspondingly, we select three subsets of the normal group dataset, each of which has the same number of users as suicidal group users. Then, these three subsets are combined with the SWOM and SWM dataset, respectively. We divide the obtained sub-datasets into two groups according to whether the suicide group data contains tree hole posts. The experimental process of these two groups of data is exactly the same.

Firstly, three sub-datasets of each group are fed into DHE-SD model. For each sub-dataset, three models with the best performance are selected, and they are used to predict the label of each sample in the test set. Then, we integrate the prediction results of these three models. Through the above operations, we obtain the prediction results of the first-level ensemble. Next, the three prediction results obtained from the first-level ensemble are integrated again. The result of the second-level ensemble is regarded as the final prediction label.

In addition, we separately combine the data of SWM and SWOM with the normal group data, and directly use the TextCNN, DPCNN and FastText models for classification. We also try to use classical oversampling and undersampling methods to deal with the problem of data imbalance, which can prove the effectiveness of our proposed DHE-SD model.

### 5.3. Evaluation Indicator

Accuracy is a commonly used as an evaluation indicator in classification tasks, defined as the proportion of samples classified correctly in the total sample. The calculation formula is:

(5) $$ACC=\frac{TP+TN}{TP+FN+FP+TN}$$

where TP represents the number of true positive samples, TN represents the number of true negative samples, FP represents the number of false positive samples and FN represents the number of false negative samples.

However, for the dataset with an imbalanced number of positive and negative samples, the accuracy indicator has some flaws. This is because even if the classifier predicts all samples as a category with the largest number of samples, it can get a high accuracy score. Nevertheless, such a classifier is actually meaningless.

The F1-score is a more reasonable evaluation indicator while the number of positive and negative samples is imbalanced, and its calculation formula is:

(6) $$F1=\frac{2\times P\times R}{P+R}$$

where *P* represents precision; *R* represents recall. They are defined as:

(7) $$P=\frac{TP}{TP+FP}$$

(8) $$R=\frac{TP}{TP+FN}$$

Since our dataset is imbalanced, we mainly use F1-score as the evaluation indicator in the experiment, and give the corresponding accuracy for reference.

## 6. Results and Analysis

In order to verify the effectiveness of the DHE-SD model, we use the two datasets introduced in Section 4 to conduct experiments, respectively.

### 6.1. Performance Comparison

We apply the three deep learning models introduced in Section 3.4 to the dataset proposed in Section 4.1, in which the suicide group data from SWOM and the results are shown in Table 4. The experimental results show that TextCNN achieves best accuracy and F1-score, FastText takes second place and DPCNN has relatively poor performance in our dataset. This may be because TextCNN uses multiple convolution kernels of different sizes for convolution, which can obtain feature representations with different granularities, so that the model has stronger representation ability. However, the number of layers of DPCNN is too large, which may cause model degradation and reduce model performance. Due to the significant gap between the distribution of positive and negative samples in the dataset, all results are affected by data imbalance.

The performance of our proposed DHE-SD model is shown in Table 5. In order to verify the effectiveness of the model, we compare it with oversampling and undersampling methods, which are commonly used methods to solve the problem of data imbalance in text classification. In oversampling, we randomly sample users in the SWOM dataset and add their posts to the experimental dataset. For undersampling, we randomly delete users in the normal group to make it consistent with the number of suicide-prone users. We choose TextCNN which has the best performance in Table 1 as baseline. It can be seen from the experimental results in Table 5 that oversampling achieves a little performance improvement. The performance of undersampling even declines; the reason may be that the sampling operation loses information partly. The DHE-SD model achieves the best performance among all methods. Compared with the baseline, accuracy and F1-score of the proposed model increase by 1.81% and 2.74%, respectively.

### 6.2. Sentence-Level Mask Analysis

When identifying suicide-prone users, using data containing tree holes is not universal. This is because most social media users would not post in tree holes. Table 6 shows the results of the three deep learning models on the SWM and normal group dataset. TextCNN still achieves the highest accuracy and F1-score, but the performance of all three models decrease significantly. The average accuracy and F1-score drop by 7.69% and 10.29%, respectively. Most of the current work uses data obtained from tree holes, but posts in tree holes usually contain words with strong emotions, and the final classification results may be overly dependent on these words. Therefore, after deleting the posts in the tree holes, the prediction performance of the model will slump.

We hope to identify users’ suicidal ideation as accurately as possible without using tree hole data. Therefore, we use the SWM and normal group data to train the DHE-SD model, and the experimental results are shown in Table 7. Because TextCNN achieves the best performance on the SWM and normal group dataset, it is regarded as the baseline. The results show that the proposed model improves in both accuracy and F1-score compared with the baseline, of which F1-score improves by more than 4%.

### 6.3. Case Study

In order to show the classification effect of the DHE-SD model more intuitively, we manually select some posts of several users, obtain the classification results of these users and compare the results of the proposed model and the best performing baseline model. The results are shown in Table 8. Users 1 and 3 are annotated with suicidal ideation, and user 2 is a normal group user.

Since user 1’s posts contain words such as ‘die’, ‘cut wrist’, ‘jump off the building’ and other words that express obvious suicidal ideation, both the baseline and DHE-SD model can accurately identify the user’s suicidal ideation. User 2 does not actually have suicidal ideation, but his/her posts express complaints about daily life, and share lyrics with significant negative emotions, which causes the baseline model to misclassify him/her as a suicide-prone individual. This is also one of the common difficulties in the field of suicide detection. Due to the combination of hierarchical ensemble strategy, the proposed model alleviates this problem to a certain extent, and then gives the correct classification results. We label user 3 as suicide-prone according to the data in the tree hole. We find that the user expressed clear suicidal ideation only in the tree hole, but hardly expressed negative emotions in other posts. In the SWM dataset, we mask all users’ tree hole posts, which makes the two models fail to identify the user’s hidden suicidal ideation correctly.

### 6.4. Discussion

The above experimental results show that, compared with the baseline model, the proposed DHE-SD model achieves the best suicide detection performance before and after applying the sentence-level mask mechanism. In addition, we also compare the DHE-SD model with the traditional methods dealing with data imbalance, such as oversampling and undersampling, to illustrate its superiority in dealing with suicide detection tasks in the case of data imbalance.

To demonstrate the effectiveness of the DHE-SD model further, we also use the public Weibo dataset introduced in Section 4.2 to conduct experiments. The experimental results in Table 9 show that oversampling and undersampling achieve 86.48% and 86.56% F1-score separately. Meanwhile, the DHE-SD model achieves the best performance. In comparison with the baseline model, accuracy and F1-score have a 2.04% and 3.75% increase, respectively. The results on our proposed and public datasets prove that the DHE-SD model outperforms existing methods on the imbalanced dataset, and can effectively detect suicide-prone users on social media.

## 7. Conclusions and Future Work

In this paper, we propose a deep suicide detection model called DHE-SD based on the hierarchical ensemble strategy, which can effectively alleviate the problem of data imbalance in the field of suicide detection. In addition, we construct a Chinese social media suicide detection dataset, and verify the effectiveness of the DHE-SD model on this dataset and another public dataset. Experiments show that the model achieves the best performance on both datasets. In addition, we propose a sentence-level mask mechanism. It can be seen from the results that after using this mechanism to delete user posts with strong suicidal ideation, the DHE-SD model can still identify suicide-prone users on social media effectively. In general, the proposed model can be used for the preliminary screening of social media suicide-prone users, and provide a basis for clinicians to review.

While there have been many suicide detection studies based on social media data, few of these studies can be put into practical application. This may because the concealment of social media makes it difficult for researchers to communicate directly with users, and the privacy policy of social media platforms greatly limits the promotion of related applications. In addition, the social media data used for research often have problems such as non-standard expression, lack of symbols and subjectivity in the annotating of experimental data by experts, which makes it difficult to apply and promote the proposed DHE-SD model in practice.

In future work, we will try to combine more information, such as constructing users’ social knowledge graph, so as to detect the suicidal ideation of social media users more effectively. We will also try our best to put our model into practical application to help more individuals with suicidal ideation.

## Figures and Tables

**Table 1:** Details of normal group data and suicidal group data before and after using sentence-level mask mechanism.

Dataset	#Users	#Posts	Avg_Post	Avg_Length
SWOM	1606	132,654	83.65	58.71
SWM	1606	98,680	62.48	55.18
Normal	2915	426,161	147.20	62.77

Note: #Users represents the number of users. #Posts represents the number of posts. Avg_Post represents the average number of posts posted by each user. Avg_Length represents the average number of characters per user’s posts. SWOM and SWM are suicide group datasets before and after using sentence-level mask mechanism. Normal represents the normal group dataset.

**Table 2:** Examples of user posts in different datasets.

Dataset	Example
SWOM	Loop this song infinitely, crying while listening. I want to see my blood pouring out from the cut arteries, and I feel my body Mom, happy holidays! I miss you so much.
SWM	Loop this song infinitely, crying while listening. He laughs really well.
Normal	Rather than want to prove that I can do anything, it’s better to admit that I am a waste. I hope my daughter is healthy and happy…

**Table 3:** Examples of high-frequency words in different datasets.

SWOM	SWM	Normal
want very all not die nothing alive pain cry hahaha	uncomfortable get off work lonely very want sorry hope love like break up	laugh see good-looking cry cute like buy cannot today feel

**Table 4:** The results of three deep learning models on the SWOM and normal group dataset.

Model	Accuracy	F1-Score
DPCNN	90.58%	86.45%
FastText	92.10%	88.33%
TextCNN	93.94%	91.18%
Average	92.21%	88.65%

**Table 5:** The results of different methods of dealing with data imbalance on the SWOM and normal group dataset.

Method	Accuracy	F1-Score
Baseline	93.94%	91.18%
Oversampling	94.40%	92.09%
Undersampling	93.67%	90.98%
DHE-SD	**95.75**%	**93.92**%

**Table 6:** The results of the three deep learning models on the SWM and normal group dataset.

Model	Accuracy	F1-Score
DPCNN	81.53% (−9.05%)	74.75% (−11.70%)
FastText	84.87% (−7.23%)	78.28% (−10.05%)
TextCNN	**87.15%** (−6.79%)	**82.04%** (−9.14%)
Average	84.51% (−7.69%)	78.36% (−10.29%)

Note: The content in parentheses represents the difference between the results of the same model when using the SWM and SWOM datasets

**Table 7:** Performance comparison of the DHE-SD model on the SWM and normal group dataset. ↑ represents the performance improvement of DHE-SD compared with baseline.

Method	Accuracy	F1-Score
Baseline	87.15%	82.04%
DHE-SD	**89.67%**	**86.15%**
	↑ 2.52%	↑ 4.11%

**Table 8:** Examples of case studies of different models.

User	Text	Label	Baseline	DHE-SD
1	I am tired with crying. Maybe I can sleep after taking more pills. Taking medicine, jumping from a building, cutting my wrists, drinking too much… I am too tired. I do not want to die anymore. I want to live well. Death is actually a relief	1	1	1
2	They are too rational and weigh the pros and cons every second. You are aphasia alone and hold on to the advanced stage of cancer. Last night, I dreamed of working overtime to collapse, and then said that I quit, fxxk, and then quit to relax… It is nice that so many people love you. Happy birthday!	0	1	0
3	The boy I love, happy 520, always healthy and happy! Fortunately, I was vaccinated. If I am unhappy, I will have a cup of milk tea. If I am not in a good mood after drinking, I will have another cup! I hope the firemen will return safely!	1	0	0

**Table 9:** The results of different methods of dealing with data imbalance on the public Weibo data.

Method	Accuracy	F1-Score
Baseline	91.07%	85.87%
Oversampling	91.16%	86.48%
Undersampling	90.93%	86.56%
DHE-SD	**93.11%**	**89.62%**

## Data Availability

Not applicable.
